# When ‘cultures of care’ meet: entanglements and accountabilities at the intersection of animal research and patient involvement in the UK

**DOI:** 10.1080/14649365.2020.1814850

**Published:** 2020-09-03

**Authors:** Richard Gorman, Gail Davies

**Affiliations:** Department of Geography, University of Exeter, Exeter, UK

**Keywords:** Care, culture of care, animal research, patient and public involvement, institutions, geographies of science, Care, culture du care, recherche sur les animaux, participation des patients et du public, institutions, géographie des sciences, Cuidado, cultura del cuidado, investigación animal, participación del público y del paciente, instituciones, geografías de la ciencia

## Abstract

A good culture of care, empowering individuals within organisations to care and reflecting wider social expectations about care, is now a well-documented aspiration in managing practices of laboratory animal research and establishing priorities for patient and public health. However, there is little attention to how different institutional cultures of care interact and what happens to the accountabilities of caring roles and the entanglements of caring practices when institutional cultures meet. Drawing on research exploring the increasing practices of patient and public involvement (PPI) within animal research in the UK, we identify three ways in which cultures of care are changing in encounters between biomedical researchers and people affected by health conditions. Firstly, patient involvement in animal research brings additional bodies to *care for* within research facilities. Secondly, patient and public groups are seen as an increasingly important group to *convey a culture of care* to. Thirdly, involvement brings opportunities for patients and publics to *connect care* for both human and animals. However, more attention is required to understand how shifts towards cultures of care distribute power and responsibility to care within institutions and at their boundaries, where responsibilities to care may be disconnected from the power to effect meaningful changes.

## RÉSUMÉ

Une culture du care de qualité, qui donne aux personnes au sein des organisations les moyens de prodiguer des soins et reflète des attentes sociales plus larges concernant la notion de care, est maintenant une aspiration bien documentée dans les pratiques de gestion de la recherche sur les animaux en laboratoire et l’établissement des priorités pour les patients et la santé publique. On accorde cependant peu d’attention à la manière dont les différentes cultures institutionnelles du care interagissent et ce qu’il advient des responsabilités des rôles de soins et des intrications des pratiques de care quand les cultures institutionnelles se rencontrent. En nous appuyant sur des recherches explorant l’augmentation de la pratique de participation des patients et du public (*patient and public involvement* – PPI) dans la recherche sur les animaux au Royaume-Uni, nous identifions trois manières dont les cultures du care sont en train de changer dans les rencontres entre des chercheurs biomédicaux et des personnes touchées par des problèmes de santé. Premièrement, la participation des patients dans la recherche sur les animaux amène plus d’êtres vivants à qui prodiguer des soins dans les centres de recherche. Deuxièmement, on considère que les groupes de patients et du public forment une cohorte à qui il est de plus en plus important de communiquer *une culture du care*. Troisièmement, cette participation donne aux patients et au public des opportunités de *lier le care* pour les humains et pour les animaux. Il faudra cependant du travail plus approfondi pour comprendre comment les changements vers les cultures du care distribuent les pouvoirs et les responsabilités dans les institutions aussi bien qu’à leurs frontières, où les responsabilités du care peuvent être déconnectées du pouvoir pour engendrer des changements significatifs.

## RESUMEN

Una buena cultura de la atención, que empodera a las personas en organizaciones hacia el cuidado y que refleja expectativas sociales más amplias sobre la atención, es ahora una aspiración bien documentada en la gestión de prácticas de investigación con animales de laboratorio y el establecimiento de prioridades para la salud pública y del paciente. Sin embargo, se presta poca atención a cómo interactúan las diferentes culturas institucionales de cuidado y qué sucede con los roles de cuidado y los enredos de las prácticas de cuidado cuando las culturas institucionales se encuentran. Basándonos en la investigación que explora las prácticas de participación del paciente y del público (PPI) dentro de la investigación con animales en el Reino Unido, identificamos tres formas en las que las culturas del cuidado están cambiando en los encuentros entre investigadores biomédicos y personas afectadas por problemas de salud. En primer lugar, la participación de los pacientes en la investigación con animales aporta organismos adicionales que cuidar dentro de las instalaciones de investigación. En segundo lugar, los grupos de pacientes y públicos se consideran un grupo cada vez más importante al que transmitir una cultura de cuidado. En tercer lugar, la participación brinda oportunidades para que los pacientes y el público conecten el cuidado tanto para humanos como para animales. Sin embargo, se requiere más atención para comprender cómo los cambios hacia las culturas del cuidado distribuyen el poder y la responsabilidad del cuidado dentro de las instituciones y en sus límites, donde las responsabilidades del cuidado pueden estar desconectadas del poder de efectuar cambios significativos.

## Encountering animal research

We start this paper with a composite narrative from our research. Two patient representatives of a medical research charity, with family experience of a rare genetic condition, are invited to an animal research facility in the UK. They are working with biomedical scientists whose research, using mice, might help them and other families who share this genetic trait. As patient advocates, their involvement can be understood as an expression of care for themselves and the future care of others. When they arrive, staff are welcoming, but uncertain how to manoeuvre one person’s wheelchair over the stepped entrance to the facility where animals are housed. The staff shift from greeting them as co-researchers to offering physical assistance as they move through biosecurity protocols. Once inside, two animal technicians, who care for the animals day-to-day, talk through the research procedures. One is enthused about meeting potential beneficiaries of their work, as it helps ameliorate the moral stress of caring for animals used in experiments; the other is less comfortable with this encounter and the possible disturbance to the mice. Towards the end of the day, at a debrief, the project researchers thank the patient representatives for their work as research collaborators, expressing hopes that they can convey to others the good culture of care they have found at the facility. At this point, it is not clear if they are talking about their culture of care for animals or their culture of care for patients, or the nature of the relationship between the two.

Our paper seeks to understand the intersection between cultures of care in animal research and patient and public involvement (PPI) in health research. It is located at the interface of two arenas in which accountability for care is increasingly structured around the idea of generating, sustaining, and conveying a good culture of care. In UK animal research, facilities are now required by regulators to create a culture of care for the animals they use through supporting staff, fostering communication, and demonstrating respectful and humane attitudes towards animals (Animals in Science Regulation Unit, 2015). Similarly, a culture of care is central to the UK’s National Health Service (NHS) service delivery, through a focus on providing good care for patients, supporting staff that care, and empowering the voice of patients and practitioners to speak out and reshape priorities for healthcare. The idea of a culture of care holds together complex institutional accountabilities – it is mandated by state regulation, generated through organisational networks, and demanding of public openness. Our opening vignette also indicates entanglements between cultures of care. Care is offered and received by facility staff, scientific researchers, and patient representatives, yet who is giving and receiving care shifts throughout the day. Focusing on what happens when the culture of care for animal research meets the culture of care around PPI reminds us that care cannot provide any guarantee of a ‘smooth harmonious world’, for it is grounded in the ‘inescapable troubles of interdependent existences’ (De la Bellacasa, 2017, p. 70), which may be located in different institutional cultures.

The central role of laboratory animals in articulating questions at the interface of animal and human health and care has echoes of Haraway’s writing, over 20 years ago, on how to think with Oncomouse^TM^ (Haraway, 1997). This transgenic mouse model was a key figure in Haraway’s provocation about how to live with the creatures we create, travelling into future writings on care, with the recognition that ‘nothing comes without its world’ (De la Bellacasa, 2017, p. 91). Yet, these worlds have changed. What was once experimental is increasingly institutionalized. Haraway’s writing bought new laboratory worlds and unfamiliar animal relations into view. Twenty years on our intimate entanglements with laboratory animal relations are increasingly being written through the regulatory guidelines (Davies et al., 2018) and professional and occupational practices (Friese & Latimer, 2019) that allocate responsibilities for care. These multi-institutional dimensions complement work on multi-species relations foregrounded in much social science work on laboratory animal care to date (Davies, 2012; Greenhough & Roe, 2019).

There are three reasons for turning now to think through entanglements between animal and human health via the cultures of care that constitute these worlds. The first is contextual. A good culture of care is increasingly central to the generation and regulation of care within diverse institutional settings, including animal research practices and the setting of healthcare priorities through PPI. A culture of care is used to describe the organisational strategies designed to encourage service providers to care about those they care for, those they work with, and the work they do. The second is conceptual. The way care is understood, performed, and valued is being reshaped by these regulatory practices. Even whilst commentators criticize the broad ambiguity (Gillin et al., 2017) and narrow instrumentalization (Goodwin, 2018) of the concept of a culture of care, it is restructuring personal responsibilities and institutional accountabilities around care. The third is conjunctural. If we are to take care seriously as a complexly situated, institutionally regulated, and performative activity – that responds to the way it is measured – challenging questions emerge around what happens to the entanglements and accountabilities of care at the boundaries between different institutional cultures.

In what follows, we first contextualize the growth of a culture of care as a regulatory object across animal and health research within the UK. Secondly, we introduce our research on what happens as PPI moves upstream to preclinical contexts and encounters biomedical research involving animals. PPI is a useful focus for tracking how concepts of a culture of care travel across biomedical and health research and for attending to changing institutional expectations around care. Thirdly, we introduce perspectives from patients, scientists, and others working at the interfaces between animal research and PPI. We explore who is caring for whom, what happens when meanings of care diverge, and how institutional accountabilities generate and constrain these exchanges. Concluding, we reflect on how shifts towards cultures of care distribute power and responsibility to care within institutions and at their boundaries.

## Caring cultures

On initial examination, cultural geographers should be well placed to locate and interrogate the growing emphasis on cultures of care. As Atkinson et al. (2011, p. 563) argue, care ‘affords geographers a richness of possibilities through which to engage critically with a range of politically charged discourses’. Our past work has drawn on care literatures to document the material entanglements, affective relations, and ethical attachments fostering care for human and non-human animals (Davies, 2012; Davies et al., 2018; Gorman, 2017). Recent geographical work considers how care stretches across scales, spaces, and temporalities (Sothern & Dickinson, 2011), and involves interdependency and multidirectionality within networks of care (Milligan & Wiles, 2010). Yet, at the same time, discussion of culture has fallen away as object of exploration and locus of explanation. The same literatures that ushered in new tools to talk about the naturecultures of laboratory animal relations also warned about the reification or blackboxing of culture (Latour, 1987) and the particular tendency of technoscience to consider itself ‘the culture of no culture’, a term from Traweek (1988), resonating throughout Haraway’s 1997 text. Medicine too, argues Taylor (2003), sees itself as a ‘culture of no culture’. As Atkinson (2002, p. 121) suggests, ‘researchers and policy makers in the field of health systems have been uneasy with the notion of the informal or the cultural as a major category of analysis’. Much current geographical research on care is not imagined through the lens of culture, but as ‘a complex network of actants and actions with multidirectional flows of activity and connections’ (Milligan, 2014, p. 1).

The resurgence of culture as the basis for care within health and biomedical research thus arrives as something of a surprise and a challenge to the trajectory of geographical analysis, though studies of nursing in critical health geography do highlight how contemporary spaces of health and care contain diverse organisational and management cultures (Andrews et al., 2005). Analysing inquiries into care failings, Goodwin (2018) notes how ‘culture’ is increasingly positioned as both a cause and explanation of failure. The ‘culture of care’ appears as the ‘culture of no culture’ fails to protect people and animals from harm: the managerial emphasis on efficiency leaves no mechanism to ensure vulnerable people can eat in ways that nurture them (Francis, 2013), whilst the streamlining ethical review to electronic platforms removes opportunities for acknowledging recurring concerns (Animals in Science Regulation Unit, 2014). The response of health and care professionals is to re-centre culture: ‘if we want to improve care, we must focus on nurturing appropriate cultures’ (West et al., 2014, p. 5). This framing of culture argues cultural change will drive improvements within an organisation. However, discussions around cultures of care risk arriving at the same problems as earlier cultural geographers (Mitchell, 1995). Ascribing responsibility for past failures and accountability for future improvements to ‘culture’ can have a reductive tendency, assuming culture is ‘a homogenous and stable entity that acts on others’ (Goodwin, 2018, p. 10), which can be re-engineered in purposeful ways.

Nevertheless, growing talk about a culture of care is an interesting shift for it recognizes the absence, or marginalising, of care within professional logics of public management in human and animal health (Hobson-West & Timmons, 2016). Despite challenges in defining a culture of care (Gillin et al., 2017), consistent themes emerge. Commonly occurring is the idea a culture of care involves going ‘beyond being compliant with applicable rules and regulations and strives to meet the full intent of established rules and regulation’ (Brown et al., 2018). Cultures of care have to be empowered to confront and transform the lack of care within extant technoscientific and managerial logics. In his independent review of patient safety, Berwick (2013) notes that ‘regulation alone cannot solve the problems highlighted’. He argues ‘culture will trump rules, standards and control strategies every single time’ (p. 11). Culture is not singular in these discussions: cultures can be caring or uncaring, top-down or bottom-up. A culture of care is not only about external standards; it is about situated, affective, and embodied labour. This growing emphasis on a culture of care opens up geographical questions about how the relational and affective networks of care are located, measured, nurtured, hindered, and rewarded within and beyond institutions. We explore these questions first through practices of animal research and then through PPI in health, before considering how caring practices are increasingly entangled with shifting accountabilities at their boundaries.

### Culture of care in animal research

Creating and sustaining a good culture of care is increasingly the focus of regulation in laboratory animal research. The concept dates from the 1990s and recently become formalized in UK regulation (Jennings & Smith, 2015, p. 42). New Zealand’s National Animal Ethics Advisory Committee (NAEAC) defined a culture of care in 2002 as a personal duty of care, which ‘involves more than the basics of animal care. It involves a genuine commitment to the welfare of the animals, a respect for the contribution they make to your work, and a desire to enhance their well-being beyond the minimum standards: in short, a culture of care’ (NAEAC, 2002). NAEAC recognized its dynamism, noting society’s expectations around animal welfare are ‘constantly evolving’ and those involved in animal care must ‘keep abreast of these changes and help constantly improve the culture of care’. This link between personal responsibility for animal welfare and responsiveness to changing societal concerns has become core to later definitions. The 3.52 million research procedures carried out on animals annually in the UK are regulated centrally by the Home Office, which is charged with ensuring responsible research and care. A good culture of care is seen as integral to this and is defined as ‘an environment which is informed by societal expectations of respectful and humane attitudes towards animals used in research’ (Animals in Science Regulation Unit, 2015, p. 4). The regulator also recognizes that ‘each establishment will have its own way of conveying its culture of care’ (Animals in Science Regulation Unit, 2015, p. 4).

Focus on the culture of care in UK animal research followed exposés at Huntington Life Sciences in 1997 and returned after allegations about research conduct at Imperial College London in 2013. The failings at Huntington Life Sciences were considered to breach animal welfare laws, resulting in individual prosecutions for animal cruelty and a move by the sector to recruit people who genuinely cared about animals. However, the Home Office review of the second instance recorded no unlawful behaviour, concluding ‘there was a widespread poor culture of care’ and calling for ‘an improved culture of care across the whole establishment’ (Animals in Science Regulation Unit, 2014, p. 7). Here, care failures were seen as institutional and systemic, including: a ‘them and us’ culture separating animal technicians from researchers, an electronic ethics system reducing discussion of reoccurring concerns, and staff moving through on short-term contracts. The regulator’s guidance was updated to make explicit responsibilities for ‘nurturing a culture of care’ (Home Office, 2014, p. 22) for animals and the people who work within establishments. It additionally charged institutional Animal Welfare and Ethical Review Bodies (AWERBs) with promoting ‘a culture of care within the establishment and, as appropriate, in the wider community’ (Home Office, 2014, p. 89).

Following the Imperial College inquiries, the idea of a culture of care has increasingly been used to make care visible to regulators, as a focus for governance within institutions, and as a means of demonstrating wider social accountability. This promotion of a culture of care within UK animal research accompanies complex shifts in the sector towards institutions being more open about their use of animals in research (McLeod & Hobson-West, 2016). Together these emphasize the complex relational nature of a culture of care (Davies et al., 2018). A culture of care can be considered as a networked assemblage of allocated responsibilities, required competencies, effective communication, and responsiveness to wider patient and public concerns. Yet opening out the idea of a culture of care in this way raises questions as to where an institutional culture of care might end and what happens at the boundaries with other establishments. Bayne and Turner (2020) argue the culture of care in animal research must extend to collaborating institutions. Their focus is scientific collaborators, but similar questions emerge at the interfaces between PPI and animal research. Until now, societal expectations around animal research have been incorporated into cultures of care in abstract ways: augmenting public trust (Klein & Bayne, 2007) or positioning publics as recipients of biomedical research who benefit from cultures of care ‘driving productivity in unprecedented ways’ (Brown et al., 2018). Where patients have been imagined in the culture of care of an animal research facility, it is as hypothetical figures, rather than individuals with relevant lived experiences (Davies, Gorman, Crudgington et al., 2020). The rise of PPI in health research brings specific questions about how communication takes places at this boundary and how this patterns who speaks of care for whom.

### Culture of care and PPI

The culture of care for patients and publics in UK health gained prominence in discussions following the ‘scandal’ of high mortality rates and poor quality care at Mid Staffordshire NHS Trust between 2005 and 2009 (Campbell, 2013). Care failings were attributed to the perpetuation of an ‘insidious, negative culture’ (Francis, 2013), which enabled ‘fear, bullying, acceptance of poor standards’, produced a ‘closed’ culture, and led to the prioritisation of business objectives over patients (Gillin et al., 2017, p. 5217). The subsequent Francis Report recommended renewed ‘focus on a culture of caring’ (Francis, 2013, p. 1695) in place of the culture of fear it found. The Culture of Care Barometer was developed to assess local cultures of care within the NHS, measuring four key parameters: the values, culture, and communication of an organisation; the support and management available within an organisation; the relationships between colleagues; and the constraints shaping individuals’ abilities to do their jobs (Rafferty et al., 2017). Yet, ambiguity remains over ‘exactly what constitutes these newly coined terminologies, and the extent of their influence on patient care’ (Gillin et al., 2017, p. 5217).

How far culture is able to achieve gains in patient care requires understanding the distribution of power within an institution and how other cultural norms have come to marginalize the work of care. It also requires a geographical perspective as care spans spatial scales from the configuration of nursing practices (Andrews et al., 2005) to organisational research priorities (Coulter, 2011). An effective culture of care is expected to permeate an institution, with all being equal participants in the co-creation of shared values and outcomes (Kawamura, 2013). This egalitarian aspiration is important to avoid concentrating pressure on certain staff, reifying the assumption care is something nurses do (Rafferty et al., 2015). To be transformative the culture of care has to sustain those already responsible for care and also change cultures that relegate care. Hesselink et al. (2013) suggest a focus on the culture of care can help value aspects of healthcare that have been overshadowed by dominant rational and process-oriented logics.

The growing notion of patient-centricity in healthcare and biomedical research has been a critical part of this shift in the logics of care (Mol, 2008). Cultures of care in PPI have received less attention than those in clinics, but there is growing recognition that ‘patients too are involved in creating health care cultures’ (Scott et al., 2003, p. 115), and that they act ‘both [as] consumers of care and as co-producers of the culture that produces that care’ (Hyde & Davies, 2004, p. 1409). Focusing on the culture of care from patient perspectives opens up discussions about whose priorities are reflected in different forms of care and whose voice matters in potential trade-offs between cure and care (Chaufan et al., 2012). A patient-centred culture of care, argue Frampton et al. (2017, p. 2), is one that ‘continuously integrates patient and family perspectives and involvement – at the point of care, in health care system design, and in defining outcomes that matter most’. This integration increasingly involves PPI in ‘upstream’ biomedical research where patient representatives can encounter questions around other cultures of care, including those within animal research. Whilst PPI originated in clinical trials development, practices of involvement have grown with aspirations for ‘patients and the public to be involved in all stages of research’ (Thornton, 2008, p. 904).

In what follows, we focus on what is being said in encounters between animal research and patient involvement. As Maccarthy et al. (2019, p. 1) note, ‘preclinical research is not a traditionally patient-facing discipline and implementing meaningful public and patient involvement (PPI) can be a serious challenge in the absence of well-defined support structures’. The exchanges below confirm this, but also show how changing relations around a ‘culture of care’ can be used to highlight what is needed to support new conversations. In tracing these, we do not suggest there is one way this should or could be done, nor try to identify what the optimal culture of care might be at the intersection of these worlds. The complexities mapped out above, and in the other papers in this special issue, caution against this. Instead, we are interested in the stories told about these encounters and the way these narratives make connections and disconnections around care (De la Bellacasa, 2017). Stories situate perspectives on care in ways that have the potential to shape caring cultures (Greenhough & Roe, 2019). In tracing who speaks from these spaces in-between institutional cultures, we are interested in who talks about care, who listens, how positions switch between caring for and caring about, and how these performances include pre-set ideas and practices of what care is, could, or should be (De la Bellacasa, 2017). These identities and expectations are shaped by the way regulatory practices and institutional guidelines allocate the work of care, yet it is only by tracing their intersection in practice that we can suggest what happens as cultures of animal and patient care meet.

## Researching animal research

This study is part of the Wellcome Trust funded Animal Research Nexus Programme, which explores the historical dimensions and social relations of animal research in the UK (Davies, Gorman, Greenhough et al., 2020). Our work at Exeter considers how the rise of PPI brings patient groups into new conversations around the practices and priorities of animal research. Formal practices of PPI – broadly defined as carrying out research in active partnership ‘with’ people affected by research – are increasingly embedded within regulatory frameworks and research systems. Funders, such as the National Institute of Health Research and medical research charities, now only fund research developed through involving patients (Wilson et al., 2015). We started research through interviewing medical research charities running PPI networks: exploring their institutional use of PPI activities around animal research, prompting reflections on intersecting practices of care and caring, and following up with the research scientists they had funded who did innovative involvement.

We interviewed 17 professionals who were supporting research involvement through roles within funding bodies, research support charities and/or medical research charities; and eleven basic and/or translational research scientists who had experience of, or were seeking opportunities for, PPI in their research. Interviews were semi-structured, giving people an opportunity to provide perspectives on the key features and challenges of involvement around animal research. Working in partnership with medical research charities, we then surveyed their involvement networks to explore the experiences of those involved in research and invited patient representatives to take part in a research interview. We interviewed 22 people who had been involved in research across different health conditions. These representatives had taken part in activities ranging from visiting scientists in laboratories to reviewing, ranking, and scoring research proposals. They were invited to reflect on these processes and how they facilitated the inclusion of their perspectives. Transcripts were returned to participants for comments and clarifications, and all respondents were given pseudonyms. Ethics approval for this research was granted by the University of Exeter.

We supplemented interviews with participant observation at patient involvement and engagement events around animal research. These signalled how organisations frame and structure these encounters, the care and labour involved in producing them, and the range of roles and expertise they involved. Building on earlier collaborative agenda-setting work with the animal research community (Davies et al., 2016), we embedded our research in ongoing deliberation with patient representatives and other stakeholders through two workshops. The first focused on developing dialogues between lay representatives across animal research and PPI to help shape research questions. The second extended these discussions by bringing together patient representatives, research scientists, involvement professionals, and others, to discuss our interim report (Gorman & Davies, 2019). Our analysis has been shaped by conversations at these workshops and surveys following events when participants were invited to provide anonymous feedback.

Working collaboratively and adopting a ‘snowball’ approach means we are researching with those scientists and organisations already thinking about how to practice involvement, but we have been able to capture tensions through data analysis and workshop dialogue. We used NVIVO to produce a thematic analysis of our data, mapping the distribution of conversations about care from different perspectives, and considering how these enact demands on who cares for and about the different participants around animal research. These situated narratives, experiences, and imaginations of care intersect, creating new accountabilities and responsibilities, though sometimes they also fail to connect new responsibilities to the power to effect meaningful change. In the next section, we discuss three ways in which cultures of care are transformed in encounters between animal research and people affected by health conditions, through extending, conveying, and connecting care, before concluding around these challenges.

## Caring in, for, and through animal research

### Extending care

In our work, we found some patient representatives valued the opportunity to act as an ‘interface’ between scientific communities and wider publics, and mobilise their family experiences to counter the disconnection from some biomedical research. Rachel describes her experience in ways that resonate with the role hoped for PPI in extending health care and research (Coulter, 2011).

I see my role as trying to look at the interface between what they [scientists] do and maybe the community, and just ask some questions that might allow them to see. There is some brilliant science […] but sometimes I think the science runs away from some of the challenges that the families face. So I see part of my role as being, “Here’s some questions that genuinely I have, and things that concern me as a parent and as a family living with this, and as scientists, how do you meet that?” Rachel (patient representative)

Yet, bringing patient representatives – physically and emotionally – into spaces associated with biomedical knowledge production also raises issues for facilities, patients, and scientists. Patients are new, and additional, bodies to care for within institutional cultures of animal research. Caring for a mouse engages different relationalities than caring for a human patient or representative – even if both are affected by a similar condition. Research organisations are having to consider how PPI activities (re)shape local cultures of care. This includes asking questions around who patient representatives meet, what animals are encountered, and how the physical and emotional aspects of engagement will be accommodated.

Scientific researchers are not discharging clinical care but still need to think about what caring for patient bodies means. Practical and physical challenges exist in opening up animal research facilities for PPI, such as how people with limited mobility can move through the biosecurity barriers that keep research animals healthy. As Ruth describes below:

We had some really interesting experiences taking patients into [research] facilities, largely because I think at one facility we had to autoclave a wheelchair. I mean just things that we’d never considered about the physicality of this kind of project and when you’ve got people with serious illnesses and trying to get them past barriers. Ruth (Research Engagement Professional, Research Support Organisation)

What is known about the unfolding of disease in animals, and thus important to their institutional culture of care, also needs to be opened up with care to visiting patient representatives and lay reviewers. Whilst the culture of care within animal research stresses the importance of being open about the harms experienced by animals and the care provided for them, bringing patients into the facility requires attuning to their distinctive knowledge and experiences. Ideas of ‘openness’ have to be carefully negotiated as there is the potential to cause harm through involvement. As Sian explains:

I brief all my students, “be very careful what you say”. Because we’ve had situations where we’ve talked about Parkinson’s and the symptoms, and we do it very pragmatically, “Parkinson’s disease is this, it causes this, in the long term you’ll get this, a third of patients get dementia”. They haven’t been told they might get dementia! We’ve been in a situation where someone’s been in the room and this patient had no idea that he had an increased risk of dementia, and that’s a huge bombshell for a patient to get. […] That’s a risk I think, with involvement, you start talking about, “this happens, and this happens”, and you talk about the way the animals die. They will take that home and keep that with them. It touches on the ethics of doing it, that there is a potential to cause harm through involvement. Sian (Biomedical Researcher, University Sector)

Doing high quality and meaningful involvement requires time, resource, and expertise in bringing patients into research spaces. Researchers working in pre-clinical contexts may not have experience of meeting and talking with people affected by the health conditions they study. Some researchers valued these exchanges for giving their work purpose. As Robert suggests: ‘it was a really valuable thing to do and it was a morale-building and enthusiasm building exercise, to have those people affected by Parkinson’s disease in the building’ Robert (Biomedical Researcher, University Sector). However, other researchers used to working with animals did not feel trained or comfortable in interacting with patients in PPI processes.

The intersection of caring for animals and patients results in new responsibilities for managers and supervisors, in ensuring researchers and technicians are supported in their encounters with patients, and can support patient representatives in turn. The need to care for patients as new bodies, as well as additional stakeholders, in these encounters destabilises ideas that animals are the default objects of care within animal research facilities. These encounters with patients are valued, but they are also narrated through stories of challenges, which indicate they go beyond the care protocols and institutional structures shaping current cultures of animal care. PPI requires extending the institutional culture of care, providing good care for patients as well as animals, and supporting the researchers and other facility staff so as to avoid the potential for harm.

### Conveying care

Patient representatives are not only recipients of a culture of care, they have become an important stakeholder group to which animal research institutions must convey and communicate their institutional culture of care to. These personal encounters can serve to alleviate anxieties patients may have about being reliant on research involving ‘intimate entanglements with the worlds of laboratory animals’ (Davies, Gorman, Crudgington et al., 2020). Learning about – and in some cases, witnessing – the culture of care in laboratory animal research did reassure some participants. As Ted and Tessa outline:

I felt that the amount of care showed for the animals; the personal attachment that people had to the work they were doing. I’d heard about it before but when you actually see it, I think it makes a bigger impression. Ted (patient representative)

The lady who showed us round, she couldn’t get through to us enough that the welfare of the animals is paramount, and it showed. She said an interesting comment, which was she didn’t employ anybody who didn’t like animals, that made me feel a little bit better, I’ll be honest. Tessa (engagement event participant)

As Tessa indicates, these cage-side encounters do emphasise practices of good animal care. Whilst this often focussed on ‘presenting’ research practices, this was directly connected and related to capacities to reshape research practices, as patient representatives learnt about, and became more confident in questioning, care, welfare, and translational research. UK animal research facilities are increasingly open, but organised involvement events understandably focus on those who want to communicate their work and high standards of care, part of what Holmberg and Ideland (2012) identify as the ‘selective openness’ of animal research. Animal research institutions are keen those taking part in lab tours and other involvement events can convey this culture of care to others by acting as ambassadors. And many patient representatives feel empowered by this and are happy to take on this role.

I’ve been able to disseminate some of the knowledge, for example, when it comes to the use of animal testing. I’ve been able to explain and talk about the control systems in place and the ethics, the most robust in the world. If anybody has any concerns, I am able to communicate that to the families and carers I know. Toby (patient representative)

As indicated earlier, the emphasis on a culture of care within animal research intersects with the openness agenda. PPI activities are often framed by recent organisational experiences of supporting openness around animal research. There is less experience of using patient voices to inform health-related research. Those reporting benefits of PPI often mentioned conveying a culture of care for animals to patients; few talked of including patient perspectives in animal research.

That’s one of the benefits of [involvement], our [patient representatives] do see the 3Rs^1^ in action and the care and affection that some of our researchers have for the animals that they use in their research, and how the Home Office regulations are applied in practice. Stephen (Research Involvement Professional, Medical Research Charity)

This is not to be cynical about potential effects on the culture of care for animals. The growth of people visiting laboratories for involvement and engagement events is said to encourage staff to showcase the facility at its best – demonstrating the idea of going ‘beyond being compliant’ that is central to a culture of care (Brown et al., 2018).

I’ve heard it kind of reflected that the animal welfare standard has in some respects improved because the facilities, there’s a much greater spotlight being shone on them now. So particularly academic institutions, there is an expectation that their animal facility will be presentable in such a way that people could come and look around it, so the institutions have become quite conscious of this. Ruth (Research Engagement Professional, Research Support Organisation)

However, whilst many patient representatives do value this access, they also gain an ‘uneasy responsibility’ in public discussions (Davies, Gorman, Crudgington et al., 2020). Involved patients have to navigate and narrate their encounters with animal research with a certain level, and duty, of care within their own health communities, and at the level of wider public discourse. Patients’ positions within networks of care change again. Patients become important intermediaries, connecting human and animal care, narrating hopeful futures and lessening fears, a role which requires careful, and caring, labour.

### Connecting care

Being invited into research facilities means some patients do see their role as contributing directly to practices of animal care and welfare and the realisation of research benefits. If a culture of care is to be informed by societal expectations, patient representatives can see themselves as connecting societal concern for animal welfare with their personal interest in realising benefits from research. The way they seek to enact these responsibilities often overlaps with other roles taken in research review, from scientific peer review, to the role of the lay member within the AWERB in UK regulation. Sometimes these research oversight responsibilities are understood formally through role specifications, as Win explains, and sometimes they are more informal, as Tabitha outlines.

That is a part of our briefing, that when there is an animal testing involved that we’ve also got to look into the welfare of the animals during the duration of the research. Win (patient representative)

I think it is almost being, not being guardians but we can look out for the animals’ interests, that’s really where I would see my involvement. And also challenging that using them was the best and most effective way of running that particular trial. Does it actually have to use animals? Tabitha (patient representative)

This shifts patient representatives’ position again, asking that they care for how research is done. PPI creates opportunities for lay members to question whether alternatives have been considered, how the 3Rs have been implemented, and whether research will be adequate to produce a meaningful translational benefit that outweighs the harms. Patient representatives, like Rachel, manage this through the regulatory languages of animal research in which they have been briefed. Yet, others, like Tiffany, seek their own vocabularies to express connections between care for humans and animals.

My questions will be how many mice and for how long and have they struck the right balance between something that’s statistically valid but to keep the numbers as low as possible? Are there alternatives that were considered? Rachel (patient representative)

I think it’s all about participants in research, whether they are animals or human beings […] it’s making sure that the guardianship I suppose, due care and attention in the treatment of any research subjects is properly exercised. Tiffany (patient representative)

Tiffany and Tabitha turn to the language of ‘guardianship’ to interpret their role as patient representatives in animal research. This language may reflect their roles as carers and guardians, but diverges in use. Tabitha reaches for this word but emphasises that they not guardians for animals, whilst Tiffany acknowledges her guardianship in helping ensure governance for all research participants. Whilst patient representatives are not doing the practical work of ‘caring for’ research animals, their affective and ethical labour is being mobilized in shaping cultures of care, and they have to negotiate actively how far they can and should ‘care about’ the animals involved in research.

Embedding PPI within animal research, whether reviewing research proposals or visiting facilities to monitor research, produces additional emotional burdens. Some patient representatives identify this as labour and struggle with the work they are asked to do. Many found the lack of a clearly structured process at the intersection of animal research and patient involvement challenging. Patient representatives want to contribute to enhancing cultures of both animal and health care. However, they find the order of review processes confusing, with grant, ethical, and patient reviews overlapping. Without an understanding of how these connect, some cannot identify their involvement as meaningful, or resist the roles they are asked to play.

I wouldn’t want to monitor anything with animals because by then, the study’s already happening and whatever I question isn’t going to change anything Tina (patient representative)

If I felt that my opinions would actually have an effect on the sort of research proposals that academic staff were putting into funding bodies, I’d be more interested in contributing than I am at the moment. Tim (patient representative)

I did make the comment that when the ethical thing is not specifically in place, essentially what you’re asking me to do is to make an ethical decision […] and I just feel that that’s the wrong way round, it’s not my job to decide on the ethics of it. Rhoda (patient representative)

When they do get involved in animal research, the roles patient representatives play are complex. They occupy formal roles as research reviewers and mentors, they informally add to the lay voices reviewing animal research, and they play a vital role in connecting the ‘collective and personal hopes and fears’ (Haraway, 1997, p. 47) involved in animal research. The multiple roles played by patient representatives mean involvement is likely to increase. The opportunity to engage with the people for whom research is being carried out can remind scientists and technicians why they do often emotionally challenging work, whilst visiting laboratories can remind people affected by health conditions that careful and hopeful work is happening. One response to our workshop indicates our research further facilitated this. When asked ‘what you might change, or do differently’ following discussions, one person wrote:

I am going to find some patients who are willing to engage with my staff and arrange a meeting. I would love for my staff to hear from the ‘end users’ of their work, but also I would love it if patients could see the hard work and motivation of our staff. (Anonymous workshop feedback)

Our tracing of the connections between cultures of care suggest this intersection has potential value, for problems around institutional care are most often found when cultures are isolated and inward-looking (Goodwin, 2018). The desire to involve patient groups highlights how cultures of care are increasingly seen as open and shared accomplishments, produced by, and producing, an entanglement of different accountabilities – and directionalities – of care. However, actively making such connections extends institutional roles and adds to individual caring responsibilities. The facility manager has to learn to care for patient visitors. The scientific researcher has to adapt research proposals and communications to include patient groups. The entanglement of patient involvement with research advocacy and accountability is especially demanding for people affected by health conditions. Patient representatives may be empowered by their location at the intersection between cultures of care, but these connections are still at the margins of institutional structures. They ask much of people who have the most at stake and often with the least power to affect change. Alongside positive experiences, we heard stories from patient representatives who questioned whether they were listened to and the extent to which changes were made. These intersecting cultures create new entanglements around care, but the current accountabilities of animal research and the lack of clear processes to incorporate patient voices limit the potential for more challenging voices to be heard.

## Conclusions

De la Bellacasa (2017) writes that ‘care is embedded in the practices that maintain webs of relationality and is always happening in between’ (p. 166). In this paper, we have sought the in-between spaces of animal research and PPI, finding in them new webs of relationality, connecting cultures of care in ways that have implications for human and non-human lives. We have demonstrated how care has become an ‘unavoidable’ part of the institutional work of animal research and PPI. Our account reveals the practical issues, mixed motivations, and uncertain structures that have to be navigated between institutional cultures of care. We suggest these are not discrete cultures, but the idea of a culture of care does provide a platform for surfacing issues around how new entanglements require new patterns of accountability. Rafferty et al. (2017) propose ‘culture of care’ has value as a concept for its performative functions and the belief that ‘culture changes by talking about it’. Importantly here, talking about care with the full range of stakeholders who constitute these emerging relations of care can help address systems that create not cultures of care, but silos of care. We suggest further study of how cultures of care are operating in situ might surface these issues in other areas of social and cultural geography, such as education and clinical care.

We have also shown it is important to attend to the intersection of cultures of care. Drawing on Parr (2003, p. 217), we can see that care is not simply ‘given’, but better conceptualised as a series of precarious ‘achievements’, mediated by a range of factors, including local cultures and institutional structures that support patients’ voices. We have seen the ways this is productive as patient representatives become empowered in relation to care and research practices. But we also note it is not necessarily rewarding, nor always comforting (De la Bellacasa, 2017). There are new demands to care for and care about the many different bodies that make up the complex worlds of animal research. Care can have a disruptive potential, one that is often forgotten in favour of reducing care to a mechanism for the smoothing out of differences (De la Bellacasa, 2017). As different cultures of care intersect, they unsettle institutional boundaries and expectations, and further work is needed to connect processes of accountability for all those entangled in these practices. The encounters between people affected by health conditions and animal research demonstrate the interdependencies of care with institutional cultures. Many of those with institutional roles – researchers, funders, animal technicians and others – are still learning how to extend accepted accountabilities for care. Patient representatives in particular come into the spaces of health research and animal research in ways that multiply their relations of care. We see patients taking up positions as mentors, acting as support networks for researchers, and community organisers for their own peers. These complicate regulatory frameworks that imagine institutionally embedded patterns of caring, instead requiring – and producing – complexly situated and relational expressions of care. There is more that geographers can do to interrogate the spatialities of care embodied in regulatory imaginaries of a culture of care and consider how they intersect with institutional practices in place.

In particular, we suggest the meeting of different cultures of care requires consideration of how responsibilities intersect with the distribution of power. There are limits to how far patients can return care to the central technoscientific and managerial logics underpinning health care and research. Re-allocating accountabilities towards patients can lock in a logic that patients should care (Callon & Rabeharisoa, 2004), a move that engages patient’s affective-ethical labour to sustain interdependent worlds (De la Bellacasa, 2017). Attempts to invest new stakeholders, like patients, with the capacities to shape care practices through initiatives like PPI, even if done carefully, can be a gesture that reifies anxieties, if not accompanied by changes empowering patients to make meaningful contributions to the things they are being asked to care for. As Haraway (1997) wrote in 1997, ‘technoscience as cultural practice’ requires an attention to the accountabilities of all ‘the subjects and objects in play’ (p. 82). Culture of care provides one platform for studying this. However, more work is needed to unpack patterns of marginality and identify strategies to enable a more equitable distribution of care capacities, responsibilities, and accountabilities. This likely requires further ethnographic work, which, given the challenges of staging these encounters needs in-depth participatory work, involving social scientists, patient representatives, scientific researchers, and animal technologists. As the language of ‘culture of care’ is increasingly embraced within policy and practice, geographers – as this special issue demonstrates – could be well placed to explore this turn to institutional culture(s) as a salve for the problems ‘regulation alone cannot solve’ (Berwick, 2013, p. 11).

## Data Availability

The datasets generated during the study are being prepared for deposit to the UK Data Archive at the end of the project and are not currently publicly available. Anonymised interview transcripts from participants who consented to data sharing are available from the corresponding author, subject to reasonable request.

## Note

1. The 3Rs here refers to efforts to replace, reduce and refine the use of animals in research.

## References

[cit0001] Andrews, G. J., Holmes, D., Poland, B., Lehoux, P., Miller, K., Pringle, D., & McGilton, K. S. (2005). ‘Airplanes are flying nursing homes’: Geographies in the concepts and locales of gerontological nursing practice. *Journal of Clinical Nursing*, 14(s2), 109–120. 10.1111/j.1365-2702.2005.01276.x 16083493

[cit0002] Animals in Science Regulation Unit. (2014). *Report of ASRU Investigation into compliance*. Home Office.

[cit0003] Animals in Science Regulation Unit. (2015) . *Identification and management of patterns of low-level concerns at licensed establishments*. Home Office.

[cit0004] Atkinson, S. (2002). Political cultures, health systems and health policy. *Social Science & Medicine*, 55(1), 113–124. 10.1016/S0277-9536(01)00213-1 12137181

[cit0005] Atkinson, S., Lawson, V., & Wiles, J. (2011). Care of the body: Spaces of practice. *Social & Cultural Geography*, 12(6), 563–572. 10.1080/14649365.2011.601238 PMC3819909 24223496

[cit0006] Bayne, K., & Turner, P. V. (2020). Animal welfare standards and international collaborations. ILAR Journal, 60 (1), 86–94. doi: 10.1093/ilar/ily024 30624646

[cit0007] Berwick, D. (2013). *A promise to learn – A commitment to act: Improving the safety of patients in England*. Department of Health.

[cit0008] Brown, M., Symonowicz, C., Medina, L. V., Bratcher, N. A., Buckmaster, C. A., Klein, H., & Anderson, L. C. (2018). Culture of care: Organizational responsibilities. In R. H. Weichbrod, G. A. (Heidbrink) Thompson, & J. N. Norton (Eds.), *Management of animal care and use programs in research, education, and testing* (2nd ed). Taylor & Francis. 29787190

[cit0009] Callon, M., & Rabeharisoa, V. (2004). Gino’s lesson on humanity: Genetics, mutual entanglements and the sociologist’s role. *Economy and Society*, 33(1), 1–27. 10.1080/0308514042000176711

[cit0010] Campbell, D. (2013). Mid Staffs hospital scandal: The essential guide. *The Guardian*. https://www.theguardian.com/society/2013/feb/06/mid-staffs-hospital-scandal-guide

[cit0011] Chaufan, C., Hollister, B., Nazareno, J., & Fox, P. (2012). Medical ideology as a double-edged sword: The politics of cure and care in the making of Alzheimer’s disease. *Social Science & Medicine*, 74(5), 788–795. 10.1016/j.socscimed.2011.10.033 22265578

[cit0012] Coulter, A. (2011). *Engaging patients in healthcare*. McGraw-Hill Education.

[cit0013] Davies, G. (2012). Caring for the multiple and the multitude: Assembling animal welfare and enabling ethical critique. *Environment and Planning D: Society and Space*, 30(4), 623–638. 10.1068/d3211

[cit0014] Davies, G., Gorman, R., & Crudgington, B. (2020). Which patient takes centre stage? Placing patient voices in animal research. In S. Atkinson & R. Hunt (Eds.), *GeoHumanities and health* (pp. 141–155). Springer. 31829542

[cit0015] Davies, G., Gorman, R., Greenhough, B., Hobson-West, P., Kirk, R. G. W., Message, R., Myelnikov, D., Palmer, A., Roe, E., Ashall, V., Crudgington, B., McGlacken, R., Peres, S., & Skidmore, T. (2020). Animal research nexus: A new approach to the connections between science, health and animal welfare. *Medical Humanities*, medhum-2019-011778. 10.1136/medhum-2019-011778 PMC7786151 32075866

[cit0016] Davies, G., Greenhough, B., Hobson-West, P., & Kirk, R. G. W. (2018). Science, culture, and care in laboratory animal research: Interdisciplinary perspectives on the history and future of the 3Rs. *Science, Technology, & Human Values*, 43(4), 603–621. 10.1177/0162243918757034

[cit0017] Davies, G., Greenhough, B. J., Hobson-West, P., Kirk, R. G. W., Applebee, K., Bellingan, L. C., Berdoy, M., Buller, H., Cassaday, H. J., Davies, K., Diefenbacher, D., Druglitrø, T., Escobar, M. P., Friese, C., Herrmann, K., Hinterberger, A., Jarrett, W. J., Jayne, K., Johnson, A. M., Wolfensohn, S., & Johnson, E. R. (2016). Developing a collaborative agenda for humanities and social scientific research on laboratory animal science and welfare. *Plos One*, 11(7), 1–12. 10.1371/journal.pone.0158791 PMC4948886 27428071

[cit0018] De la Bellacasa, M. P. (2017). *Matters of care: Speculative ethics in more than human worlds*. University of Minnesota Press.

[cit0019] Frampton, S. B., Guastello, S., Hoy, L., Naylor, M., Sheridan, S., & Johnston-Fleece, M. (2017). Harnessing evidence and experience to change culture: A guiding framework for patient and family engaged care. *NAM Perspectives*, 7(1), 1–38. 10.31478/201701f

[cit0020] Francis, R. (2013). *Report of the mid Staffordshire NHS foundation trust public inquiry*. Department of Health.

[cit0021] Friese, C., & Latimer, J. (2019). Entanglements in health and well-being: Working with model organisms in biomedicine and bioscience. *Medical Anthropology Quarterly*, 33(1), 120–137. 10.1111/maq.12489 30811681 PMC6492009

[cit0022] Gillin, N., Taylor, R., & Walker, S. (2017). Exploring the concept of “caring cultures”. *Journal of Clinical Nursing*, 26(23–24), 5216–5223. 10.1111/jocn.13858 28425629

[cit0023] Goodwin, D. (2018). Cultures of caring: Healthcare ‘scandals’, inquiries, and the remaking of accountabilities. *Social Studies of Science*, 48(1), 101–124. 10.1177/0306312717751051 29316861

[cit0024] Gorman, R. (2017). Therapeutic landscapes and non-human animals: The roles and contested positions of animals within care farming assemblages. *Social & Cultural Geography*, 18(3), 315–335. 10.1080/14649365.2016.1180424

[cit0025] Gorman, R., & Davies, G. (2019). Patient and public involvement and engagement (PPIE) with animal research. https://animalresearchnexus.org/sites/default/files/patient_and_public_involvement_and_engagement_ppie_with_animal_research.pdf

[cit0026] Greenhough, B., & Roe, E. (2019). Attuning to laboratory animals and telling stories: Learning animal geography research skills from animal technologists. *Environment and Planning D: Society and Space*, 37(2), 367–384. 10.1177/0263775818807720 32655205 PMC7322828

[cit0027] Haraway, D. J. (1997). *Modest_Witness@Second_Millennium. FemaleMan©_Meets_OncoMouseTM*. Routledge.

[cit0028] Hesselink, G., Kuis, E., Pijnenburg, M., & Wollersheim, H. (2013). Measuring a caring culture in hospitals: A systematic review of instruments. *BMJ Open*, 3(e003416), 1–10. 10.1136/bmjopen-2013-003416 PMC3787470 24065697

[cit0029] Hobson-West, P., & Timmons, S. (2016). Animals and anomalies: An analysis of the UK veterinary profession and the relative lack of state reform. *The Sociological Review*, 64(1), 47–63. 10.1111/1467-954X.12254

[cit0030] Holmberg, T., & Ideland, M. (2012). Secrets and lies: “Selective openness” in the apparatus of animal experimentation. *Public Understanding of Science*, 21(3), 354–368. 10.1177/0963662510372584 23045886

[cit0031] Home Office. (2014). *Guidance on the Operation of the Animals (Scientific Procedures) Act 1986*.

[cit0032] Hyde, P., & Davies, H. T. O. (2004). Service design, culture and performance: Collusion and co-production in health care. *Human Relations*, 57(11), 1407–1426. 10.1177/0018726704049415

[cit0033] Jennings, M., & Smith, J. (2015). *A resource book for lay members of ethical review and similar bodies worldwide*. RSPCA.

[cit0034] Kawamura, K. M. (2013). Understanding the concept of care in cross‐cultural settings. *Cross Cultural Management: An International Journal*, 20(2), 100–123. 10.1108/13527601311313373

[cit0035] Klein, H. J., & Bayne, K. A. (2007). Establishing a culture of care, conscience, and responsibility: Addressing the improvement of scientific discovery and animal welfare through science-based performance standards. *ILAR Journal*, 48(1), 3–11. 10.1093/ilar.48.1.3 17170491

[cit0036] Latour, B. (1987). *Science in action: How to follow scientists and engineers through society*. Harvard Univ. Press.

[cit0037] Maccarthy, J., Guerin, S., Wilson, A. G., & Dorris, E. R. (2019). Facilitating public and patient involvement in basic and preclinical health research. *PLoS ONE*, 14(5), 1–16. 10.1371/journal.pone.0216600 PMC6516642 31086377

[cit0038] McLeod, C., & Hobson-West, P. (2016). Opening up animal research and science–society relations? A thematic analysis of transparency discourses in the United Kingdom. *Public Understanding of Science*, 25(7), 791–806. 10.1177/0963662515586320 26009149 PMC5036072

[cit0039] Milligan, C. (2014). Geographies of care. In W. Cockerham, R. Dingwall, & S. Quah (Eds.), *The wiley-blackwell encyclopedia of health, illness, behavior, and society*. Wiley-Blackwell.

[cit0040] Milligan, C., & Wiles, J. (2010). Landscapes of care. *Progress in Human Geography*, 34(6), 736–754. 10.1177/0309132510364556

[cit0041] Mitchell, D. (1995). There’s no such thing as culture: Towards a reconceptualization of the idea of culture in geography. *Transactions of the Institute of British Geographers*, 20(1), 102. 10.2307/622727

[cit0042] Mol, A. (2008). *The logic of care: Health and the problem of patient choice*. Routledge.

[cit0043] NAEAC. (2002) . *A culture of care: A guide for people working with animals in research testing and teaching*. National Animal Ethics Advisory Committee.

[cit0044] Parr, H. (2003). Medical geography: Care and caring. *Progress in Human Geography*, 27(2), 212–221. 10.1191/0309132503ph423pr

[cit0045] Rafferty, A. M., Philippou, J., Fitzpatrick, J. M., & Ball, J. (2015). *Culture of care barometer: Report to NHS England on the development and validation of an instrument to measure ‘culture of care’ in NHS trusts*. King’s College.

[cit0046] Rafferty, A. M., Philippou, J., Fitzpatrick, J. M., Pike, G., & Ball, J. (2017). Development and testing of the ‘culture of care barometer’ (CoCB) in healthcare organisations: A mixed methods study. *BMJ Open*, 7(e016677), 1–8. 10.1136/bmjopen-2017-016677 PMC5629717 28821526

[cit0047] Scott, T., Mannion, R., Marshall, M., & Davies, H. (2003). Does organisational culture influence health care performance? A review of the evidence. *Journal of Health Services Research & Policy*, 8(2), 105–117. 10.1258/135581903321466085 12820673

[cit0048] Sothern, M., & Dickinson, J. (2011). Repaying the gift of life: Self-help, organ transfer and the debt of care. *Social & Cultural Geography*, 12(8), 889–903. 10.1080/14649365.2011.624192

[cit0049] Taylor, J. S. (2003). Confronting “culture” in medicine’s “culture of no culture”. *Academic Medicine*, 78(6), 555–559. 10.1097/00001888-200306000-00003 12805033

[cit0050] Thornton, H. (2008). Patient and public involvement in clinical trials. *BMJ*, 336(7650), 903–904. 10.1136/bmj.39547.586100.80 18436920 PMC2335270

[cit0051] Traweek, S. (1988). *Life times and beamtimes: The world of high energy physicists*. Harvard University Press.

[cit0052] West, M., Eckert, R., Steward, K., & Pasmore, B. (2014). *Developing collective leadership for health care*. The King’s Fund.

[cit0053] Wilson, P. M., Mathie, E., Keenan, J., McNeilly, E., Goodman, C. M., Howe, A., Poland, F., Staniszewska, S., Kendall, S., Munday, D., Cowe, M., & Peckham, S. (2015). ReseArch with patient and public involvement: A realist evaluation - the RAPPORT study. *Health Services and Delivery Research*, 3(38), 38. 10.3310/hsdr03380 26378332

